# Supplementary material for: SCD5 Regulation by VHL Affects Cell Proliferation and Lipid Homeostasis in ccRCC
Source: Cells. 2023 Mar 8;12(6):835. doi: 10.3390/cells12060835 (PMC10047146; doi:10.3390/cells12060835)

Supplementary Figure S1

(a)

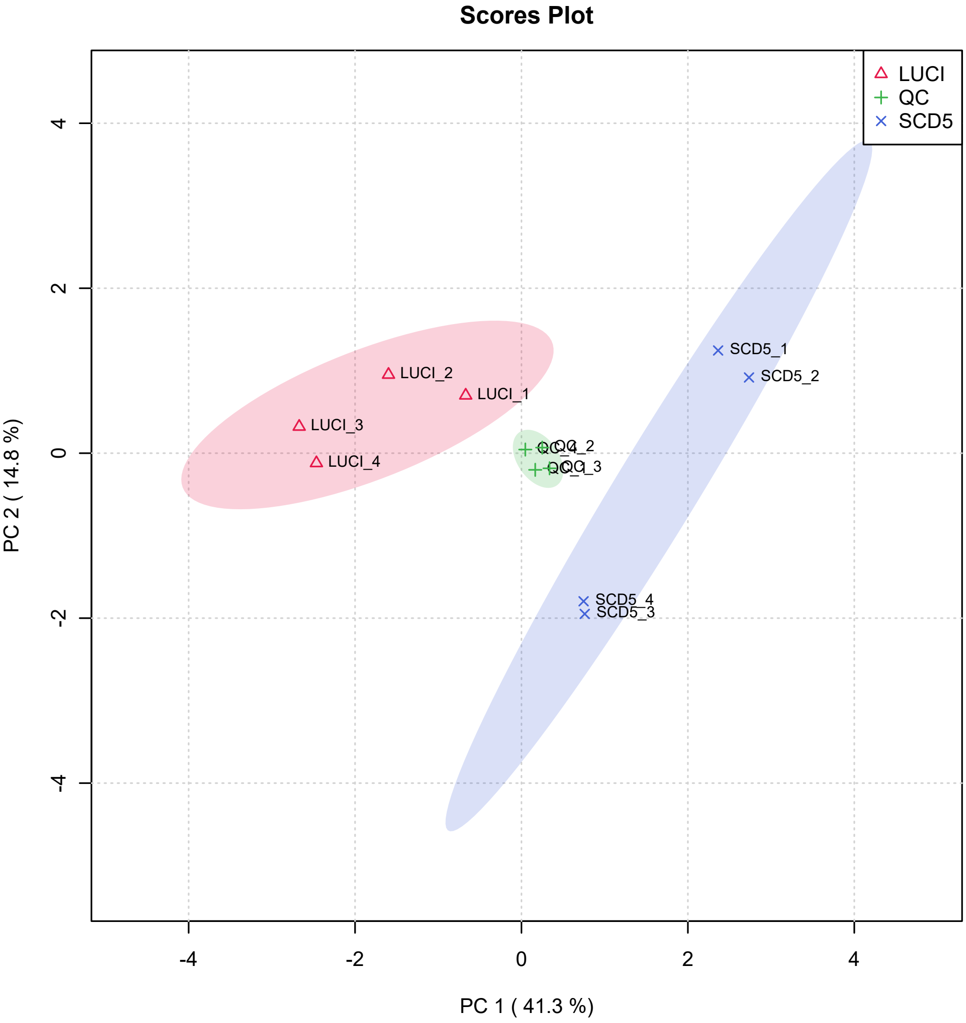

(b)

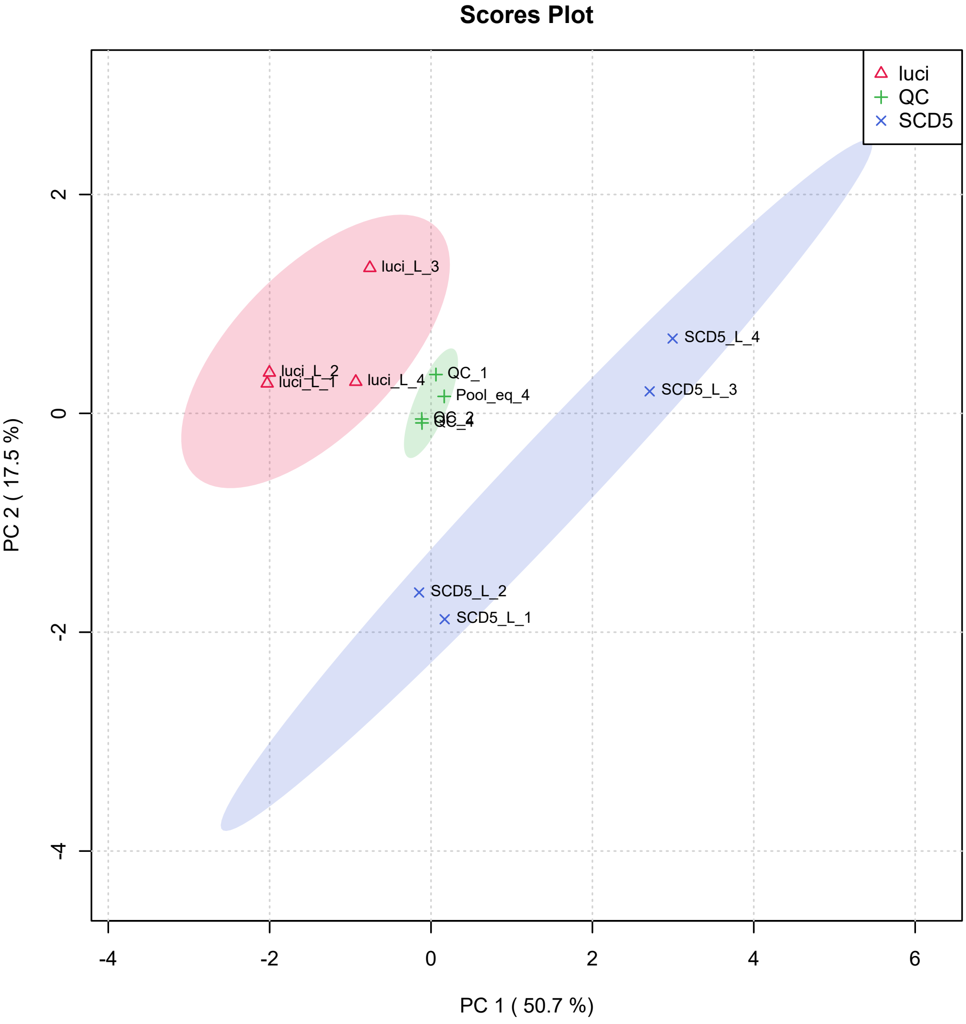

Supplementary Figure S2: Uncropped Western blots including replicates for quantitative analyses

Figure 1d

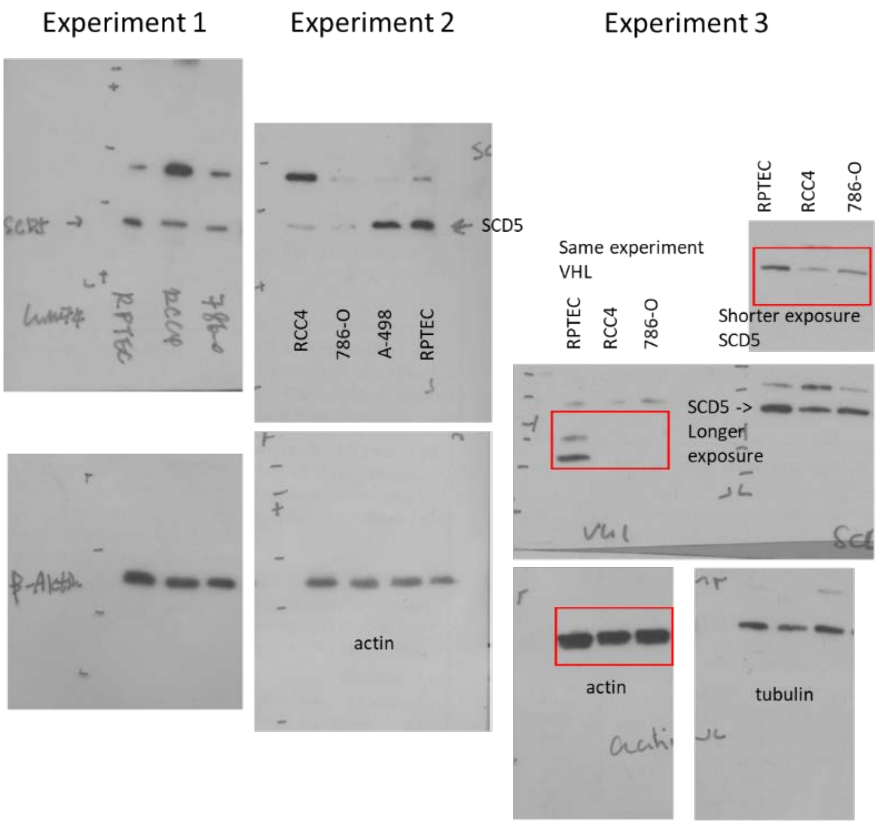

Figure 2b

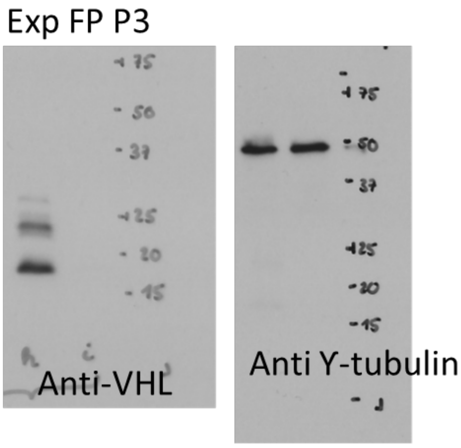

Figure 2e

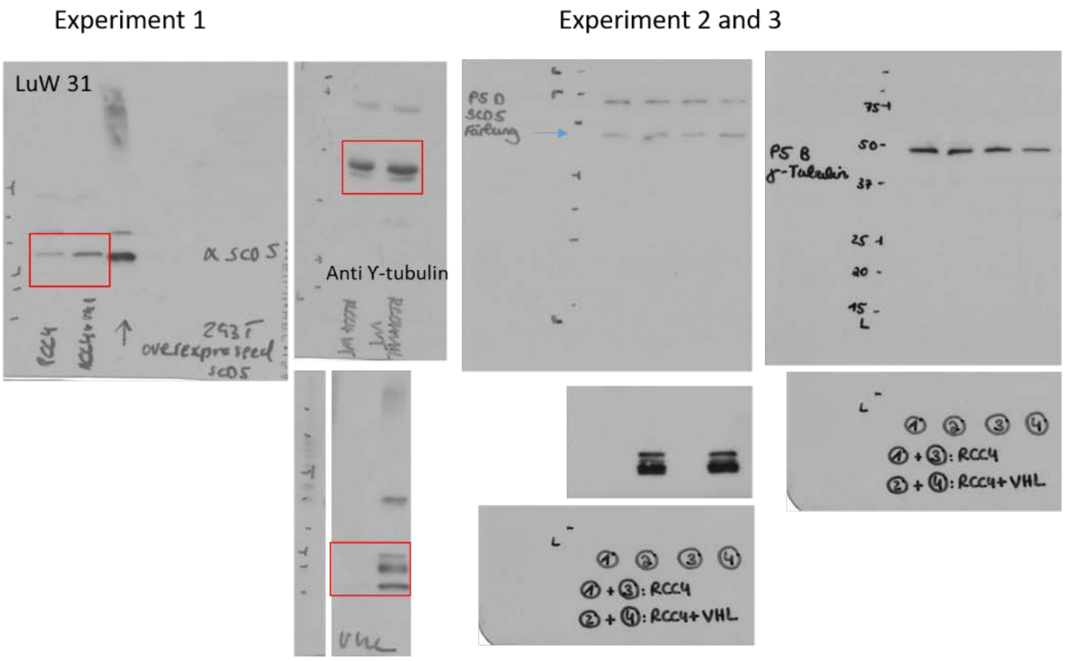

Figure 4b

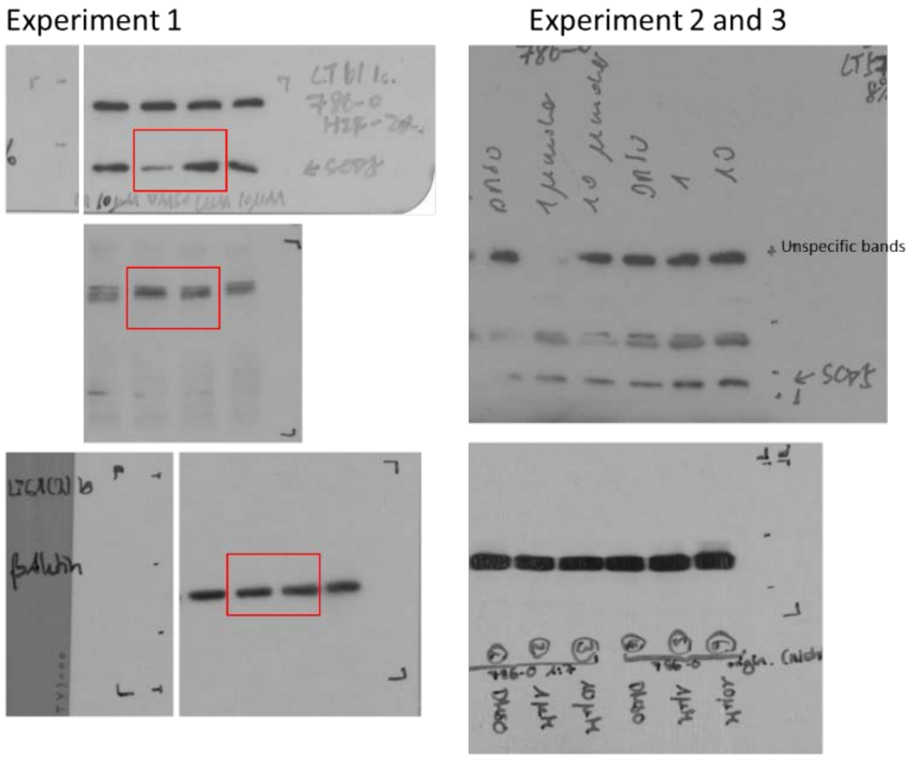

Figure 4c

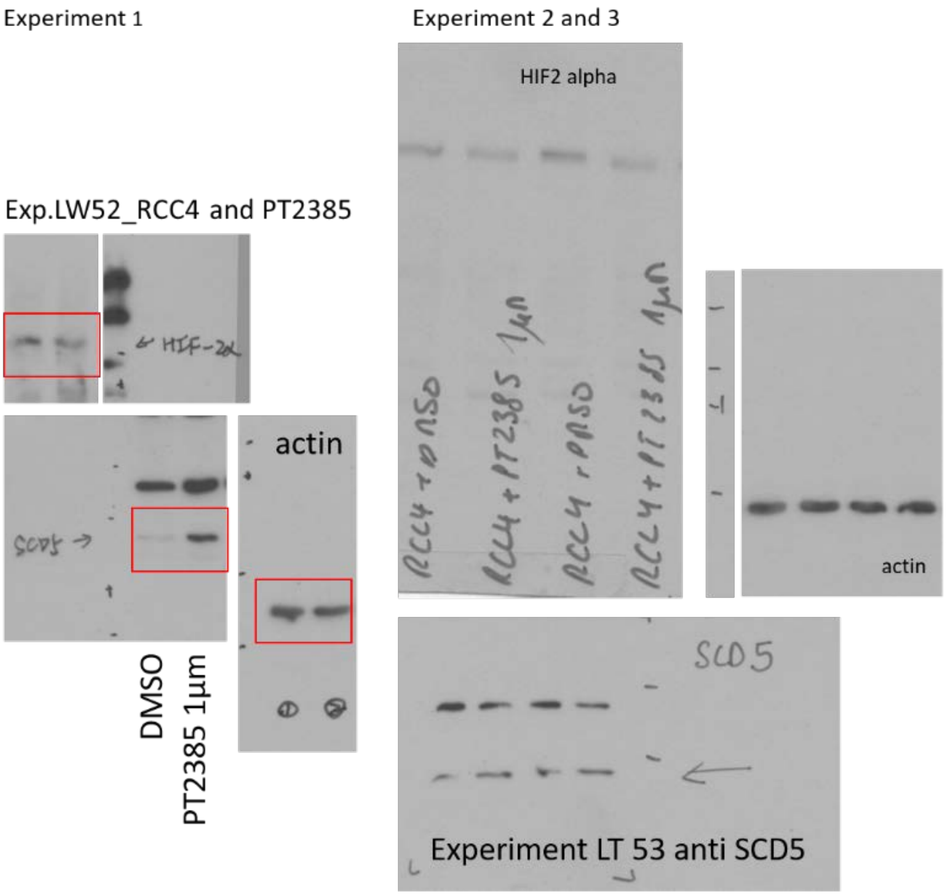

Figure 4d

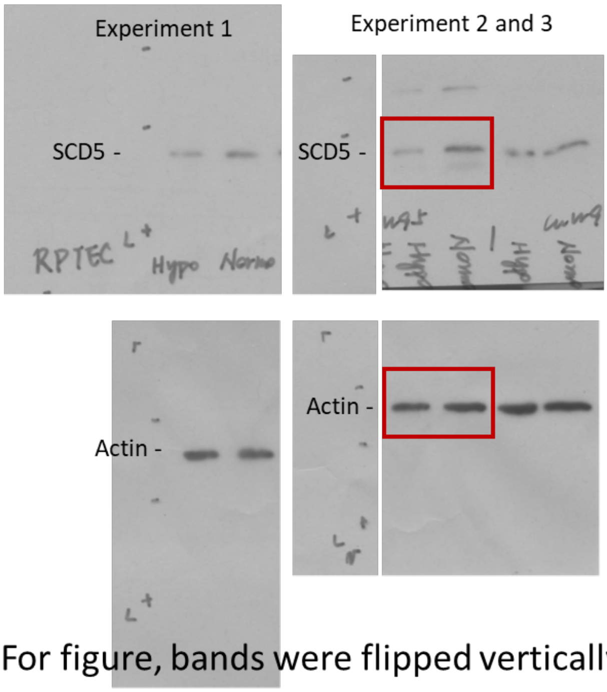

For figure, bands were flipped vertically

Figure 5b and Figure 5d

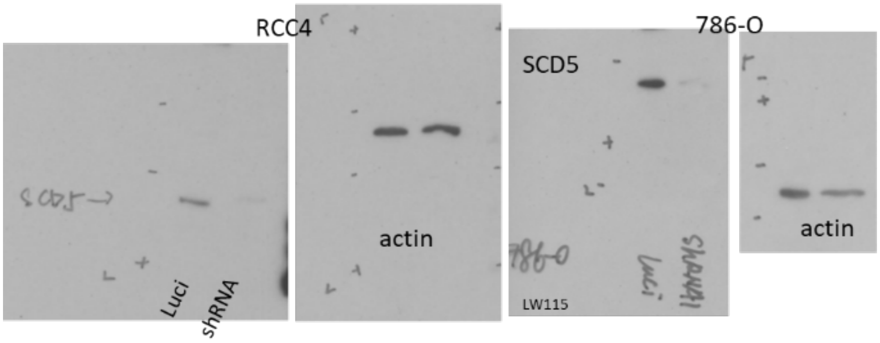

Supplement: Supplementary file 1 [file cells-12-00835-s001.zip › cells-2250021-supplementary.pdf]
